# The Role of Self-Assembled Monolayers in the Surface Modification and Interfacial Contact of Copper Fillers in Electrically Conductive Adhesives

**DOI:** 10.1021/acsami.3c14900

**Published:** 2023-12-19

**Authors:** Shanda Wang, Zhaoxia Zhou, Athanasios Goulas, Gary W. Critchlow, David C. Whalley, David A. Hutt

**Affiliations:** † Wolfson School of Mechanical, Electrical and Manufacturing Engineering, Loughborough University, Loughborough, Leicestershire LE11 3TU, U.K.; ‡ Department of Materials, Loughborough University, Loughborough, Leicestershire LE11 3TU, U.K.

**Keywords:** self-assembled monolayer, electrical interconnection, oxidation protection, copper, isotropic conductive adhesive, interface analysis, surface modification, conduction mechanisms

## Abstract

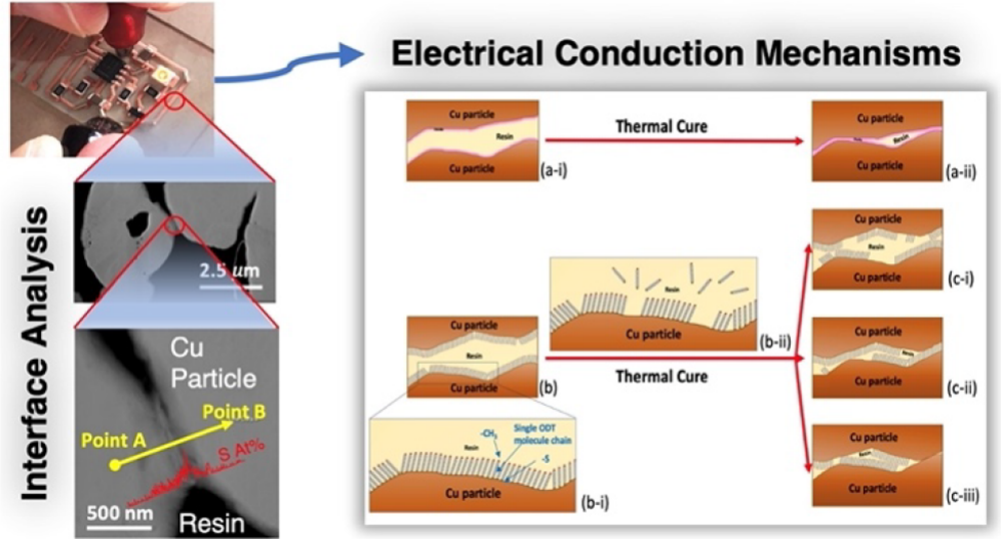

Printing of electrical circuits and interconnects using isotropic conductive adhesives (ICAs) is of great interest due to their low-temperature processing and compatibility with substrates for applications in sensors, healthcare, and flexible devices. As a lower cost alternative to silver (Ag), copper (Cu)-filled ICAs are desirable but limited by the formation of high-resistivity Cu surface oxides. To overcome this limitation, self-assembled monolayers (SAMs) of octadecanethiol (ODT) have been demonstrated to reduce the oxidation of micrometer-scale Cu powder particles for use in ICAs. However, the deposition and function of the SAM require further investigation, as described in this paper. As part of this work, the stages of the SAM deposition process, which included etching with hydrochloric acid to remove pre-existing oxides, were studied using X-ray photoelectron spectroscopy (XPS), which showed low levels of subsequent Cu oxidation when ODT coated. The treated Cu powders were combined with one- or two-part epoxy resins to make Cu-ICAs, and the effect of the Cu surface condition and weight loading on electrical conductivity was examined. When thermally cured in an inert argon atmosphere, ICAs filled with Cu protected by ODT achieved electrical conductivity up to 20 × 10^5^ S·m^–1^, comparable to Ag-ICAs, and were used to make a functional circuit. To understand the function of the SAM in these Cu-ICAs, scanning and transmission electron microscopy were used to examine the internal micro- and nano-structures along with the elemental distribution at the interfaces within sections taken from cured samples. Sulfur (S), indicative of the ODT, was still detected at the internal polymer–metal interface after curing, and particle-to-particle contacts were also examined. XPS also identified S on the surface of cured Cu-ICAs even after thermal treatment. Based on the observations, electrical contact and conduction mechanisms for these Cu-filled ICAs are proposed and discussed.

## 1 Introduction

Electrically conductive adhesives (ECAs) have gained much attention in electronics manufacturing due to their low-temperature processing, ease of use, and ability to be selectively deposited or printed into patterns.^1−4^ They are used to provide electrically conductive joints, in applications such as the attachment of flip-chip and surface-mount technology components to flexible and printed circuit boards, but can also be formulated as electrically conductive inks (ECIs) to manufacture circuit traces for printed electronics.^5−10^ Their rheology can be adjusted so that they may be screen or stencil-printed or otherwise dispensed directly onto substrates.

Isotropic conductive adhesives (ICAs) and ECIs are composite materials where a polymeric adhesive matrix binds together electrically conductive fillers. The conductive fillers in ICAs are usually silver (Ag), gold (Au), copper (Cu), or carbon-based materials.^1−4^ In most ICAs, micrometer-scale fillers are used, where the contacts between them are primarily physical in nature. Good electrical conduction is achieved through the inclusion of a high volume fraction of the filler, which must be above the percolation threshold to ensure a large number of particle-to-particle contacts.^3,4,11^ The contacts between filler particles should also be of low electrical resistance.^11^ This is in contrast to nanoparticle inks, where sintering of particles is usually achieved,^7,12,13^ although hybrid materials have also been developed.^14−17^

Ag fillers have been extensively used in ICAs due to their low electrical resistivity.^1−4,10,18^ In particular, Ag forms a surface oxide that is electrically conductive^2,4,19^ and therefore maintains a low resistance network of particles even when it has been exposed to the air. Ag fillers also have good resistance to corrosion, which is necessary for high reliability. However, the high cost of Ag limits the further application of these materials, especially for high production volumes, and finding substitute fillers has become a significant industrial requirement. The much lower cost of Cu, combined with a similarly high bulk conductivity, has attracted attention as a promising filler material.^16,20−34^ However, the surface of untreated Cu readily oxidizes when exposed to air, forming a high-resistivity layer, which prevents good electrical contacts between particles.^27−29^ To enable the use of Cu as a conductive filler in ICAs, methods to remove any existing surface oxide and prevent or inhibit oxide regrowth are required. To this end, some researchers have used Cu particles coated with other metals, such as Ag,^25−27,29,30,33^ successfully improving the conductivity and reliability of the Cu-filled ICAs. Alternatively, modification of the Cu particle surfaces with organic acids,^31^ silane coupling agents,^16,28^ and other protective coatings have been reported as methods to prevent reoxidation.^21,32^

The methodology used in the present study is to first remove the oxide from Cu powder and then apply a protective self-assembled monolayer (SAM) coating of alkanethiol, specifically octadecanethiol (ODT).^22,23,35−38^ These SAMs have been shown to create a coating that can significantly reduce the rate of oxidation of the Cu.^35,39−41^ The ODT-SAM is thought to provide a single-molecule-thick hydrophobic layer that limits the diffusion of both water vapor and oxygen to the Cu particle surface and therefore leads to a substantial decrease in the rate of reoxidation.^35,39−41^ Applying this approach to Cu powder has shown that, once the SAM has been applied, there is only a very low level of residual oxide present and the powder can be stored for many months in a freezer before being mixed with an adhesive resin, under normal laboratory conditions, to make an ICA.^22,23,36,37^ These SAM-coated Cu-filled ICAs, when cured under an argon atmosphere, have been shown to have similar initial electrical conductivity to Ag-filled materials, although their long-term reliability has been found to be lower.^22,23,36^ In a recent study, alkanethiol SAMs have also been used to supplement other surface treatments for the preservation of Cu flakes for use in conductive pastes.^34^

To further develop and utilize SAM-coated Cu within ICAs, greater understanding of the deposition process, function and fate of the SAM during the preparation and thermal curing of the materials is needed, along with any role it plays in electrical conduction. This work therefore has investigated in greater detail the process steps in the application of an ODT-SAM to the surface of commercially available micrometer-scale (average size in the range 14–25 μm) Cu powder. The processing of Cu first involved hydrochloric acid etching of the untreated powder to remove the original surface oxide, followed immediately by deposition of the ODT-SAM from an ethanol solution. X-ray photoelectron spectroscopy (XPS) was used as the primary method of assessment of the effectiveness of the process steps in controlling surface oxidation and to confirm the formation of the SAM. ICAs were subsequently prepared from these modified Cu powders (Cu-ICAs), which were stencil-printed and thermally cured. Both one- and two-part epoxies were investigated as the matrix for these Cu-ICAs, and their electrical performance was compared to that of a commercial Ag-filled ICA (Ag-ICA). This research also investigated the influence of the Cu surface condition, filler weight loading, and curing atmosphere on the ICA electrical conductivity. The purpose of the research was primarily to improve the understanding of the role played by the ODT-SAM and the mechanism of electrical conduction within the cured ICA, rather than to optimize the ICA formulation. A microstructural and surface composition analysis of the interfaces between adjacent Cu particles and between the Cu particles and the epoxy resin within the cured ICA was therefore undertaken using transmission electron microscopy (TEM). Based on the findings, this paper proposes electrical conduction mechanisms for the SAM-coated Cu-filled ICAs to assist their further study and navigate routes to improve them for future applications.

## 2 Experimental Methods

### 2.1 Materials

Generally spheroidal copper (Cu) powder with an average diameter specified in the range 14–25 μm (Supporting Information S1 shows the particle size distribution), 1-octadecanethiol [CH_3_(CH_2_)_17_SH] (ODT), and glacial acetic acid [CH_3_COOH] were all purchased from Sigma-Aldrich Ltd., UK. Concentrated hydrochloric acid 32% (HCl) and absolute ethanol [CH_3_CH_2_OH] were both purchased from Fisher Scientific, UK. EPO-TEK H20E (uncured two-part epoxy resin containing silver flake filler^42^) and EPO-TEK 353ND (uncured two-part unfilled epoxy resin^43^) were manufactured by Epoxy Technology, inc. A proprietary unfilled one-part epoxy resin was also obtained from ESL Europe Ltd., Reading, UK.

### 2.2 ODT-SAM Deposition on Copper Powder

**Figure 1:**
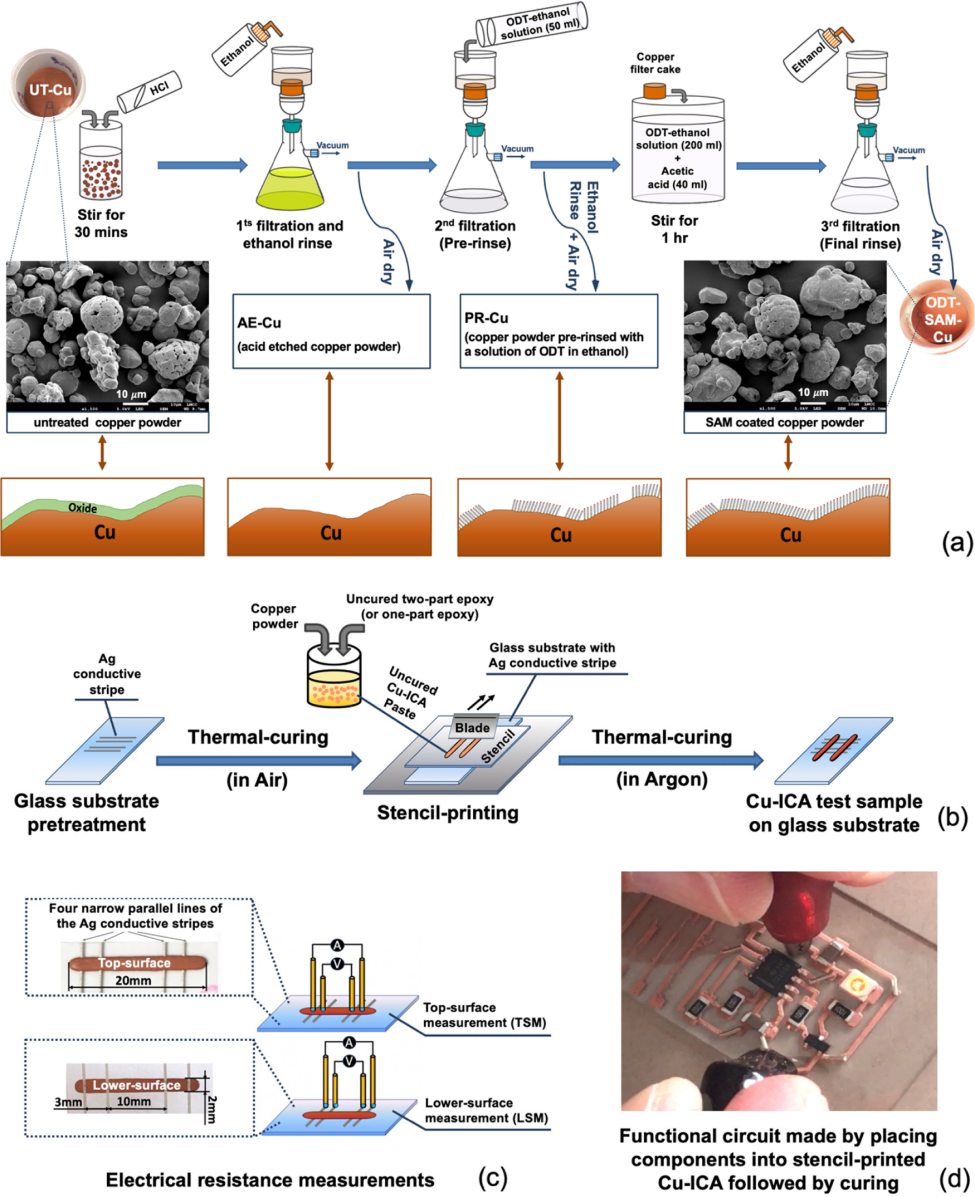
Schematic illustrations of (a) the process of the ODT-SAM coating of copper powder and (b) the preparation and printing of ICA sample tracks. (c) Top surface measurement (TSM) and lower surface measurement (LSM) electrical resistance characterization methods. (d) A printed circuit prepared using SAM-coated copper powder and one-part resin.

Figure 1a illustrates the process for surface treatment of the Cu powder investigated in this study.^22,23,36,37^ A solution of ∼0.3 g of ODT in ethanol (∼250 mL) was prepared, assisted by magnetic stirring. A quantity (50 g) of as-received, untreated Cu powder (UT-Cu) was first etched by magnetic stirring in ∼100 mL of concentrated (32%) HCl for 30 min at room temperature to remove the oxide layer on its surface. The mixture of Cu powder and HCl was then poured into a vacuum filtration system and rinsed thoroughly with 300–400 mL of ethanol ensuring that the powder was not allowed to dry at any point (1st filtration). As the last of the ethanol rinse was about to be filtered through the powder bed, a small quantity of the ODT-ethanol solution (50 mL) was added to the filter funnel and allowed to drain through the Cu powder cake for a few minutes before being filtered off (2nd filtration). This prerinse with ODT solution provided some preprotection against reoxidation. Acetic acid (40 mL) was added to the remaining 200 mL of ODT-ethanol solution. The Cu powder filter cake was briefly dried and transferred immediately to the ODT-ethanol-acetic acid solution (240 mL), and this mixture was then magnetically stirred for 1 h to allow the full formation of the ODT-SAM. This mixture was then poured into the vacuum filtration system and again thoroughly rinsed with 300–400 mL of ethanol to remove the excess thiol and acetic acid (3rd filtration). Finally, the filtered Cu powder was transferred to a clean open top glass container and dried for around 1.5 h at room temperature to obtain ODT-SAM-Cu. After preparation, the ODT-SAM-Cu powders were stored in a freezer at around −18 °C, (typically for up to 30 days) before their incorporation into ICAs in the ambient environment. Furthermore, to investigate the effect of the process steps, samples of powder were also taken and dried in air after the initial HCl etch and rinse stage (forming AE-Cu) and after the ODT-SAM solution prerinse (forming PR-Cu).

### 2.3 Preparation, Printing, and Curing of ICAs

Details of the formulations and curing process of the Ag-ICA and Cu-ICAs comprising Cu powders, either in the untreated (UT), acid-etched (AE), or ODT-coated form, combined with one- and two-part epoxies, and the notations used in this paper to identify them, are presented in Table 1. In general, the notation used is based on the following parts: metal filler composition, i.e., Cu or Ag (the Cu surface condition is indicated in brackets if this is not ODT-SAM, or not 85.7 wt %); resin type, i.e., one- (1 pt) or two-part (2 pt) epoxy; curing atmosphere, i.e., Arg (argon) or Air. For example, Cu1ptArg-ICA represents an ICA made of ODT-SAM-coated Cu powder filler (at the standard 85.7 wt % loading) with one-part epoxy resin that was cured in an argon atmosphere. To make the Cu-ICAs (Figure 1b), one- or two-part epoxy resin was combined with the appropriate Cu powder (either UT-, AE-, PR-, or ODT-SAM- Cu). A Cu-to-resin weight ratio of 6:1 (85.7 wt %) was used in this work, unless specified otherwise, with a typical batch size of 7 g. The components of the two-part epoxy resin were combined following the manufacturer’s guidelines. Mixing was carried out using a FlackTek Inc. DAC 150 FVZ-K dual asymmetric centrifugal SpeedMixer, typically for 3 min at 2500 rpm. To make the Ag-ICA, equal amounts by weight of EPO-TEK H20E parts A and B were combined and mixed using the SpeedMixer.

**Table 1:** Formulation and Curing Conditions of Different Types of ICAs Made for Trials

**sample notation**	**filler type (filler abbreviation)**	**filler composition in uncured and cured ICA** (wt %)	**resin type (resin abbreviation)**	**curing atmosphere**	**curing temperature and time**
**Ag2ptAir-ICAs**	**commercial Ag flake-filled two-part epoxy resin**(42)			**air**	**150 °C, 20 min**
**Cu2ptArg-ICAs** batch examples 1, 2, 3	**ODT-SAM-coated copper (ODT-SAM-Cu)**	**85.71 (uncured) 85.71 (cured)**	**two-part epoxy resin (2 pt)**(43)	**argon (Ar)**	**150 °C, 20 min**
**Cu1ptArg-ICAs** batch examples 1, 2, 3	**ODT-SAM-coated copper (ODT-SAM-Cu)**	**85.71 (uncured) 90.91 (cured)**	**one-part epoxy resin (1 pt)**	**argon (Ar)**	**125 °C, 10 min, and then 150 °C, 20 min**
**Cu1ptAir-ICAs**	**ODT-SAM-coated copper (ODT-SAM-Cu)**	**85.71 (uncured) 90.91 (cured)**	**one-part epoxy resin (1 pt)**	**air**	**125 °C, 10 min, and then 150 °C, 20 min**
**Cu(UT-Cu)1ptArg-ICAs**	**untreated Cu (UT-Cu)**	**85.71 (uncured) 90.91 (cured)**	**one-part epoxy resin (1 pt)**	**argon (Ar)**	**125 °C, 10 min, and then 150 °C, 20 min**
**Cu(AE-Cu)1ptArg-ICAs**	**acid-etched Cu (AE-Cu)**	**85.71 (uncured) 90.91 (cured)**	**one-part epoxy resin (1 pt)**	**argon (Ar)**	**125 °C, 10 min, and then 150 °C, 20 min**
**Cu(79)1ptArg-ICAs**	**ODT-SAM-coated copper (ODT-SAM-Cu)**	**78.95 (uncured) 86.21 (cured)**	**one-part epoxy resin (1 pt)**	**argon (Ar)**	**125 °C, 10 min, and then 150 °C, 20 min**

Standard soda-lime glass microscope slides (76 mm × 25 mm × 1 mm, Fisher Scientific, UK) were used as the substrates onto which the ICAs were printed through a brass stencil (aperture size approximately 20 mm × 2 mm and thickness approximately 300 μm) using a hand-held blade. The Ag-ICAs were stencil-printed directly onto the glass substrates, but for the Cu-ICAs, the substrates were preprepared with additional electrically conductive contacts by dispensing four narrow lines of the Ag-ICA using a Häcker Automation OurPlant XTec micro assembly system that were then cured using the standard thermal profile. The Cu-ICA samples were then stencil-printed across the top of these cured Ag tracks, at an angle of 90 deg to them, thereby enabling contact to be made with the underside of the Cu-ICA for electrical resistance measurements (Figure 1b).^36^ The Cu-ICAs were usually thermally cured on a hot plate inside a glovebox connected to an argon (Ar) supply.^37^ Ar was flowed through to create a low-oxygen environment (oxygen level <2000 ppm) for the curing process before commencing heating, which was verified with an oxygen analyzer (Z210 Oxygen Analyzer, Hitech Instruments). For the two-part-resin Cu-ICAs and Ag-ICAs, curing was carried out for 20 min at 150 °C, although the Ag-ICAs were cured in air rather than Ar. To cure the one-part resin Cu-ICAs, these were initially heated for 10 min at 125 °C and then the temperature was increased to 150 °C for a further 20 min.

### 2.4 Heat-Treatment of SAM-Coated Copper Powder (HT-SAM-Cu)

Two sets of ODT-SAM-Cu powder were placed in glass jars and then heat-treated under Ar (<2000 ppm of O_2_) on a hot plate at 150 °C, inside the glovebox, for 20 min (to simulate similar curing conditions of the two-part ICAs) or for 60 min. The glass jars were then sealed to maintain an Ar atmosphere inside. These HT-SAM-Cu samples were prepared for microstructure and XPS characterization with only 30 min exposure to air, to minimize any reoxidation.

### 2.5 Characterization Methods

#### 2.5.1 X-Ray Photoelectron Spectroscopy (XPS) Analysis

Surface analysis of the Cu powder and cured ICAs was carried out using a Thermo Scientific K-Alpha X-ray photoelectron spectrometer. Al Kα X-rays were used as the source, with a 400 μm spot size on the sample ensuring that as many as 1500 particles were analyzed. Standard XPS parameters for collecting survey spectra were 200 eV pass energy, 1 eV step size, 10 ms dwell time, and 10 scans. Standard XPS parameters for high-resolution (HR) spectra were 50 eV pass energy, 0.1 eV step size, 50 ms dwell time, and 5 scans. Since S is an important element in the SAM, but with only a low signal intensity, 10 HR scans were averaged for the S 2p region. Charge correction (based on the binding energy of the C–C bond at 284.8 eV) was applied to all spectra before data analysis in Avantage software. Each XPS spectrum was peak-fitted based on the possible chemical states, using standard peak binding energies characteristic of the element(s) anticipated.^44,45^

#### 2.5.2 Cross-Section Analysis

The morphologies of the Cu powder samples and of the top surface of the cured ICA samples (filler dispersion and microstructure) were characterized using a Zeiss 1530VP field emission gun scanning electron microscope (FEG-SEM). Elemental mapping was also conducted within the FEG-SEM using energy-dispersive X-ray spectroscopy (EDS). Samples of Cu powder and ICAs printed on glass were attached to carbon adhesive tape mounted on SEM stubs and coated with a thin layer of gold/palladium (Au/Pd) alloy (80/20) using a Quorum Q150R S sputter coater. The Au/Pd coating applied was sufficiently thin to not be recognized in the images. The cross-sectional microstructures of the ICAs were prepared and characterized using a focused ion beam (FIB-SEM) system (FEI Nanolab 600 Dual Beam). A platinum (Pt) layer (∼2 μm thick) was deposited on the surface of the sample prior to FIB milling to preserve the outermost surface.

A standard lift-out procedure using FIB-SEM was followed to prepare the lamella for TEM to achieve a thickness of ∼200 nm. The nanostructure of the ICA sample sections was then studied using an FEI Tecnai F20 scanning transmission electron microscope (STEM) equipped with Oxford Instruments energy-dispersive X-ray spectroscopy (EDS) with a windowless detector (X-Max^N^ 80 TLE).

#### 2.5.3 Electrical Characterization

The cross-sectional profile of a selection of the Cu-ICA tracks was measured using a Talysurf CLI 2000 surface topography instrument. The height of the ICA tracks above the glass substrates was measured at intervals of 5 μm across the width of the track, and 20 of these profiles were recorded at intervals of 0.5 mm along a 1 cm length of each sample. The average cross-sectional areas of a batch of cured sample tracks were measured this way and used to calculate the electrical conductivity of ICAs from their resistance measurements.

The electrical resistance of the ICA samples was measured with a Keithley 580 micro-ohmmeter using the four-point Kelvin probe method (Figure 1c). For all samples, contact was made with the top surface of the printed track with the voltage probes spaced 10 mm apart (top surface measurement, TSM) and, where available, contact was also made to the preprinted Ag tracks underneath the Cu-ICAs to obtain the lower surface measurement (LSM) (Figure 1c). Three measurements were made for each ICA sample track, and an average was taken. These were then used to calculate the overall average for each sample group.

## 3 Results

### 3.1 Electrical Conductivity of ICAs

**Figure 2:**
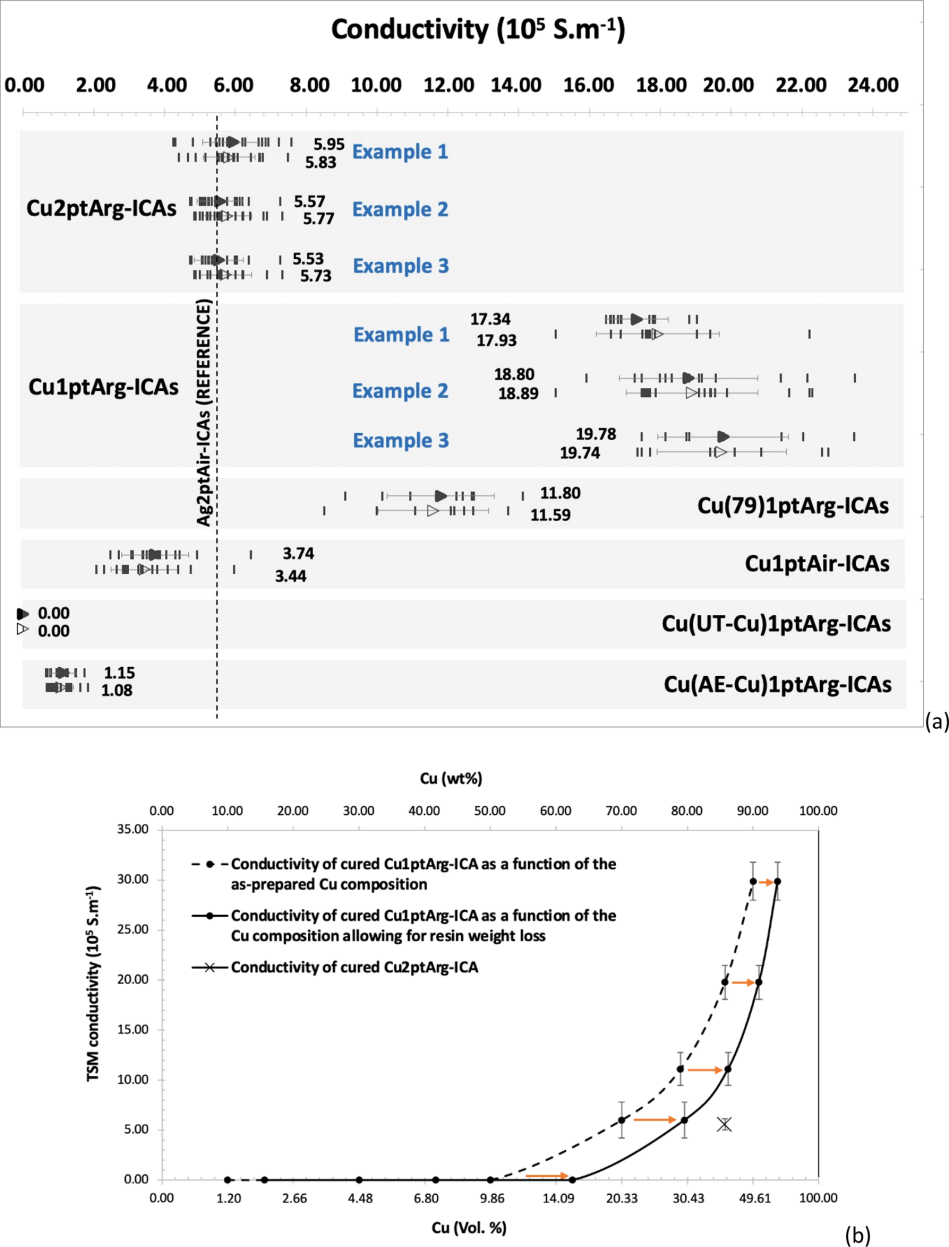
(a) Electrical conductivity distributions and average TSM (upper data sets) and LSM (lower data sets) conductivities for Cu-ICAs. The conductivity values of each individual sample track are represented by the separate markers (short lines) with the averages of the group represented by open (for LSM) or filled (for TSM) triangles and written alongside each data set. Standard deviation bars are shown as well. (b) Electrical conductivity of thermally cured Cu1ptArg-ICAs plotted against the Cu composition (volume and weight %) in the as-prepared ICA formulation and expected content allowing for resin weight loss during thermal curing (data points are the average TSM conductivity of several ICA tracks). The volume and weight fraction of Cu2ptArg-ICAs are shown for comparison, for which there was no noticeable change on curing.

The distribution and variability of the top surface measurement (TSM) and lower surface measurement (LSM) (Figure 1c) conductivities for all Cu-ICA groups are presented in Figure 2. To demonstrate repeatability, the results for different batches of the same nominal formulation are shown as Example 1, Example 2, etc. (Figure 2a). For comparison, a vertical dashed line representing the average conductivity of the standard two-part Ag-ICA is included. Overall, the average value of the conductivity of the Cu2ptArg-ICAs was similar to those of the Ag2ptAir-ICAs (5.70 ± 0.56 × 10^5^ S·m^–1^), while the conductivities of the Cu1ptArg-ICAs were noticeably higher. A similar trend was noted in earlier work using a different one-part resin^22,37^ and was also seen in Ag-filled ICAs.^46^ The average TSM and LSM conductivities were similar within the same group of samples, showing that good contact was made with the top of the printed tracks as well as through the underlying Ag tracks. Other researchers have noted the potential for the formation of corrosion at the Cu–Ag interface, which may affect conduction; however, this is usually associated with damp environments, which were not present in this work.^4^ Variations in the conductivity of the samples within a batch were thought to be due to the manual sample printing process leading to variability in the track cross section and potentially also inclusion of air pockets or voids.

The consistently lower conductivities of the Cu2ptArg-ICAs compared to those of the Cu1ptArg-ICAs are thought to be partly due to differences in the percentage of the Cu filler content in these two types of samples after curing. Although both types of ICAs were prepared with the same initial weight percentage of Cu, during cure of the Cu1ptArg-ICAs there was some weight loss (approximately 10 wt % of the ICA), attributed to losses from the one-part resin component, such that the final cured material had a significantly higher Cu content. In contrast, the Cu2ptArg-ICAs showed much less weight loss (approximately 0.2 wt % on curing). To investigate this, the one-part epoxy was mixed with different weight percentages of ODT-SAM-Cu and then thermally cured. Figure 2b shows the conductivity of the cured Cu1ptArg-ICAs plotted against their nominal, as-prepared Cu content based on the initial formulation and for the higher Cu content that would be expected after thermal curing, due to the partial one-part resin weight loss that took place. In both cases, the conductivity values are the same but are plotted against the two different wt % (or vol %) values. To create one-part Cu-ICAs with nearly the same thermally cured Cu content as the Cu2ptArg-ICA (85.71 wt % Cu), only 79 wt % Cu was needed to form Cu(79)1ptArg-ICA that gave TSM and LSM conductivities of 11.80 ± 1.51 × 10^5^ and 11.59 ± 1.56 × 10^5^ S·m^–1^, respectively (Figure 2a). These were lower values than for the typical 85.71 wt % Cu1ptArg-ICA samples but were also still more conductive than the Cu2ptArg-ICAs made with 85.71 wt % Cu. It was therefore not possible to account for the difference in the two epoxy systems through the final Cu weight loading alone.

Other aspects of the Cu powder treatment and thermal curing atmosphere were investigated to determine their influence on conductivity. The effect of the ODT-SAM-Cu powder storage time in air in the freezer before use was investigated (Supporting Information, Section S2). Storage for 30 days showed a very small reduction in the conductivity and marginal variation in the material surface composition. Similarly, it was found that samples of the ODT-SAM-Cu powder stored in the freezer for over 1000 days still produced Cu-ICAs with high conductivity. Cu(UT-Cu)1ptArg-ICAs were made using untreated (as-received) copper powder (Figure 1a) and had very high resistances, i.e., higher than the 200 kΩ capability of the micro-ohmmeter, which is represented as zero conductivity in Figure 2a. Cu(AE-Cu)1ptArg-ICAs were also made using Cu powder that had been treated with hydrochloric acid to remove the surface oxide and rinsed with ethanol, but with no SAM deposited (Figure 1a). These powders were used to make ICAs shortly after preparation, and the conductivity of the cured materials was much lower than that of the equivalent Cu1ptArg-ICAs. The standard curing process for the Cu-filled ICAs was carried out in an inert Ar environment; however, Cu1ptAir-ICAs were also made using the same batch of ODT-SAM-Cu and cured using the same thermal profile in air. The conductivities of these Cu1ptAir-ICAs were also much lower than for the Cu1ptArg-ICAs, indicating that neither the resin nor the ODT-SAM can stop the Cu particles from reoxidizing under these curing conditions, and the use of an inert atmosphere to carry out the thermal curing of the Cu-ICAs is therefore necessary.

In the literature, other groups have investigated Cu-filled ICAs. For example, Hong et al.^34^ demonstrated a flexible, air cured, highly conductive (13.4 × 10^5^ S·m^–1^) Cu-filled paste, while reliable Cu-filled ICAs with conductivity around 2.7 × 10^5^ and 1.33 × 10^5^ S·m^–1^ have also been reported using silane coupling agents.^16,28^ Chen et al.^31^ also obtained a conductivity of 22.2 × 10^5^ S·m^–1^ for Cu flake-filled ICAs using organic acids as a protective coating. Many of these are similar in performance to ICAs with Ag-coated Cu fillers^29,30^ that routinely achieve conductivity in the region of 5 × 10^5^ S·m^–1^. The conductivity values seen in the present study for Cu1ptArg-ICAs thermally cured in an inert atmosphere compare favorably with these published works; however, those cured in air (Cu1ptAir-ICAs) do not perform as well in many cases. Further investigation of the long-term reliability of the SAM preserved Cu-filled ICAs is also required for full comparison with the studies reported in the literature.

To demonstrate the effectiveness of the Cu-ICAs for printed electronics applications, a fully functional circuit was prepared, as shown in Figure 1d. This 555 timer-based circuit was produced by first stencil printing a circuit pattern of Cu(90)1pt-ICA onto a glass slide (in this case, a higher Cu loading was used to ensure a good print pattern definition). Standard surface-mount electrical components were then placed onto the uncured adhesive, and the entire assembly was then thermally cured in Ar following the one-part resin profile. Connecting power to the device resulted in the LED illuminating on and off, indicating the correct operation of the circuit. The success of this approach demonstrates both the circuit pattern formation and attachment of components in one process step, which is a significant manufacturing advantage.

### 3.2 Surface Analysis

**Figure 3:**
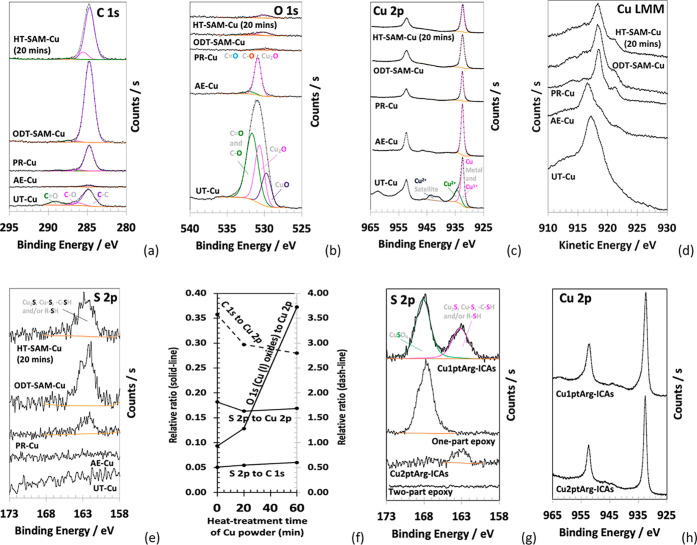
(a–e) HR XPS spectra of UT-Cu, AE-Cu, PR-Cu, ODT-SAM-Cu, and HT-SAM-Cu (150 °C for 20 min under argon) powders. (f) The influence of heat-treatment time at 150 °C under argon on the ODT-SAM-Cu powder surface composition (note, the C 1s-to-Cu 2p ratio corresponds with the right-hand axis). (g, h) HR XPS spectra of cured one- and two-part epoxy and cured one- and two-part Cu-ICAs.

High-resolution (HR) XPS spectra for samples of the copper powders at different stages of treatment, i.e., UT-Cu (untreated), AE-Cu (acid-etched), PR-Cu (prerinsed with ODT-ethanol solution), and ODT-SAM-Cu (ODT-SAM coated) (Figure 1a), are presented in Figure 3a–e. These include the Cu 2p, Cu LMM (Cu Auger), O 1s, C 1s, and S 2p regions, which were focused on identification of the presence of any Cu oxides and key elements of the ODT-SAM. In addition to these elements, traces of chlorine (typically <1.5 At%) were detected in most samples before and after treatment, possibly due to retention of some Cu chlorides in the pores of the powder. The detection of S in the ODT-SAM-Cu sample, but not in the UT-Cu, confirms the presence of ODT after the SAM treatment (Figure 3e). Furthermore, the S 2p peak position at a binding energy (BE) of 162–163 eV was similar to that previously reported for Cu-thiol bonds,^35,44,47^ indicating bonding of the ODT to the Cu surface. Compared to the UT-Cu, the C content increased significantly in the ODT-SAM-Cu, producing a very symmetrical C 1s peak (Figure 3a) at ∼284.68 eV, which is characteristic of the CH_2_ groups of the methylene chain and terminal CH_3_ of the ODT molecule.^35,44,47^ In the O 1s region (Figure 3b), the O level also showed a substantial reduction after SAM deposition compared to the UT-Cu, indicating both that much of the initial copper oxide was removed and that the SAM coating inhibited the Cu from reoxidation when exposed to the air. The Cu 2p spectra also showed significant changes, with a broad Cu 2p_3/2_ peak, at around 932 eV, and additional features around 940 eV, which are together characteristic of Cu(II) oxide in the UT-Cu.^35,44,48^ However, these were replaced with a single sharp Cu 2p_3/2_ peak after SAM deposition (ODT-SAM-Cu) (Figure 3c). According to the Cu LMM Auger peak shape,^35,44,48^ the spectrum of UT-Cu included features typical of Cu_2_O (Cu(I) oxides), CuO (Cu(II) oxides) and metallic Cu, while the Auger peaks indicated primarily metallic Cu in the ODT-SAM-Cu (Figure 3d).^48^ Multilayers of ODT on the Cu particle surfaces are not expected to form, since the SAM was applied on a largely oxide free surface.^47,49,50^

To investigate the stages of the SAM deposition process, Figure 3a–e also includes spectra from Cu powder that underwent etching with hydrochloric acid but was then only rinsed with ethanol and dried (AE-Cu) (Figure 1a). The AE-Cu did not show the presence of any S, as expected, and the C 1s peak was also very small. It is clear from the spectra that the etching process significantly reduced the features associated with Cu oxides compared to UT-Cu. However, after only around 1–2 h exposure to air after preparation, the oxide level was significantly higher than that of ODT-SAM-Cu. The PR-Cu spectra in Figure 3a–e are taken from a sample that had been etched with hydrochloric acid and then, after rinsing with ethanol, was briefly rinsed (approximately 2 to 3 min) with a solution of ODT in ethanol, rinsed with ethanol, and then dried. The PR-Cu spectra show clear features associated with the ODT-SAM including S 2p (at around 162–163 eV) and C 1s (at ∼284.6 eV). Zhang et al.^51^ measured the rate of adsorption of ODT on Cu from μM concentration solutions and found very rapid (<100 s) initial coverage, followed by a slower rearrangement and densification phase. This would indicate that an initial incomplete coverage of ODT was achieved during the PR-Cu preparation time, but despite this, it remained largely oxygen free after transferring it to the XPS chamber. These results demonstrate the importance of applying this initial ODT-SAM protection to the Cu powder, while it is still undergoing rinsing, without exposing it to air or allowing it to dry out, and is in line with earlier studies of the protection of Cu from oxidation.^35,39,40^

To investigate the conditions that the ODT-SAM-Cu would experience during thermal curing within an ICA, and to determine if the SAM would remain on the Cu particle surfaces under these conditions, samples were heated to 150 °C under Ar for 20 min to obtain HT-SAM-Cu (20 min). Figure 3a–e also shows the XPS spectra obtained from these samples. The change of the Cu 2p and Cu LMM regions of the HT-SAM-Cu samples showed that this short heat-treatment did not significantly alter the surface state of the Cu particles, with no noticeable features of Cu(I) oxide. The S 2p peak remained, albeit reduced a little in size, indicating the retention of some ODT or Cu_2_S.

In another study, ODT-SAM-Cu samples were heated for 20 and 60 min at 150 °C in Ar. Peak fitting was used to determine the change of atomic percentage indicated by the S 2p, Cu 2p, and O 1s (Cu_2_O peak) spectra, before and after heat-treatment. The relative ratios as a function of heat-treatment time are summarized in Figure 3f. The O 1s (Cu_2_O peak only) to Cu ratio increased in the heat-treated powder samples with longer heat-treatment time. The presence of O is probably due to damage to the SAM caused by the heat-treatment, which led to more facile oxidation of the Cu when exposed to the ambient environment for a short time (around 30 min) in advance of the XPS characterization. In this case, the sample with a longer heat-treatment was more easily reoxidized in the air, due to greater loss of the SAM coating. It can be concluded that some of the ODT-SAM was lost from the Cu particle surfaces after the heat-treatment of the Cu-ICAs, due to desorption of complete molecules^52^ and/or degradation of the SAM by C–S bond scission.^53,54^

The surface compositions of the cured Cu-ICAs were also analyzed using XPS and compared to those of samples of the epoxy resins cured under the same conditions but without Cu filler (Figure 3g,h). Due to the relatively large (400 μm) spot size used for XPS, the spectra from the Cu-ICAs were a hybrid of the resin and any Cu particles exposed at the surface. Figure 3h shows an intense characteristic peak of Cu(0) and Cu(I) for both of the Cu-ICAs, indicating that the epoxy resins did not leave a thick layer or residue covering the Cu particles after curing. Only small satellite features at around 943 eV were seen in the Cu 2p spectra, with the one-part epoxy resin showing a higher peak, indicating greater oxidation as compared to the two-part material (similar to the heat-treated powder data presented above). It can therefore be concluded that very little Cu(II) oxide formed during the curing process, with the overall shape of the Cu 2p spectra for the Cu-ICAs being very similar to that for ODT-SAM-Cu. Like the heat-treated Cu powder discussed above, the oxidation of the Cu within the ICA may have occurred due to exposure to low levels of O within the Ar atmosphere during curing. However, it is considered more likely that the curing process damaged the ODT layer such that oxidation occurred during exposure to the air between curing and XPS analysis.

For samples of the cured epoxy resins without any Cu filler, there were no XPS peaks due to S for the two-part resin, but peaks indicative of oxidized S species were noted for the one-part material (Figure 3g). In the Cu2ptArg-ICA, a S peak around 163 eV was observed, with no oxidized S species present. This S must have therefore originated from the ODT-SAM-Cu filler, demonstrating that some S remained on the exposed Cu particle surfaces after curing. A similar peak was observed for Cu1ptArg-ICA, indicative of thiol-Cu bonding in addition to that of the oxidized S from the surrounding resin (∼168 eV).

### 3.3 SEM and FIB Investigation of Cured ICA Samples

**Figure 4:**
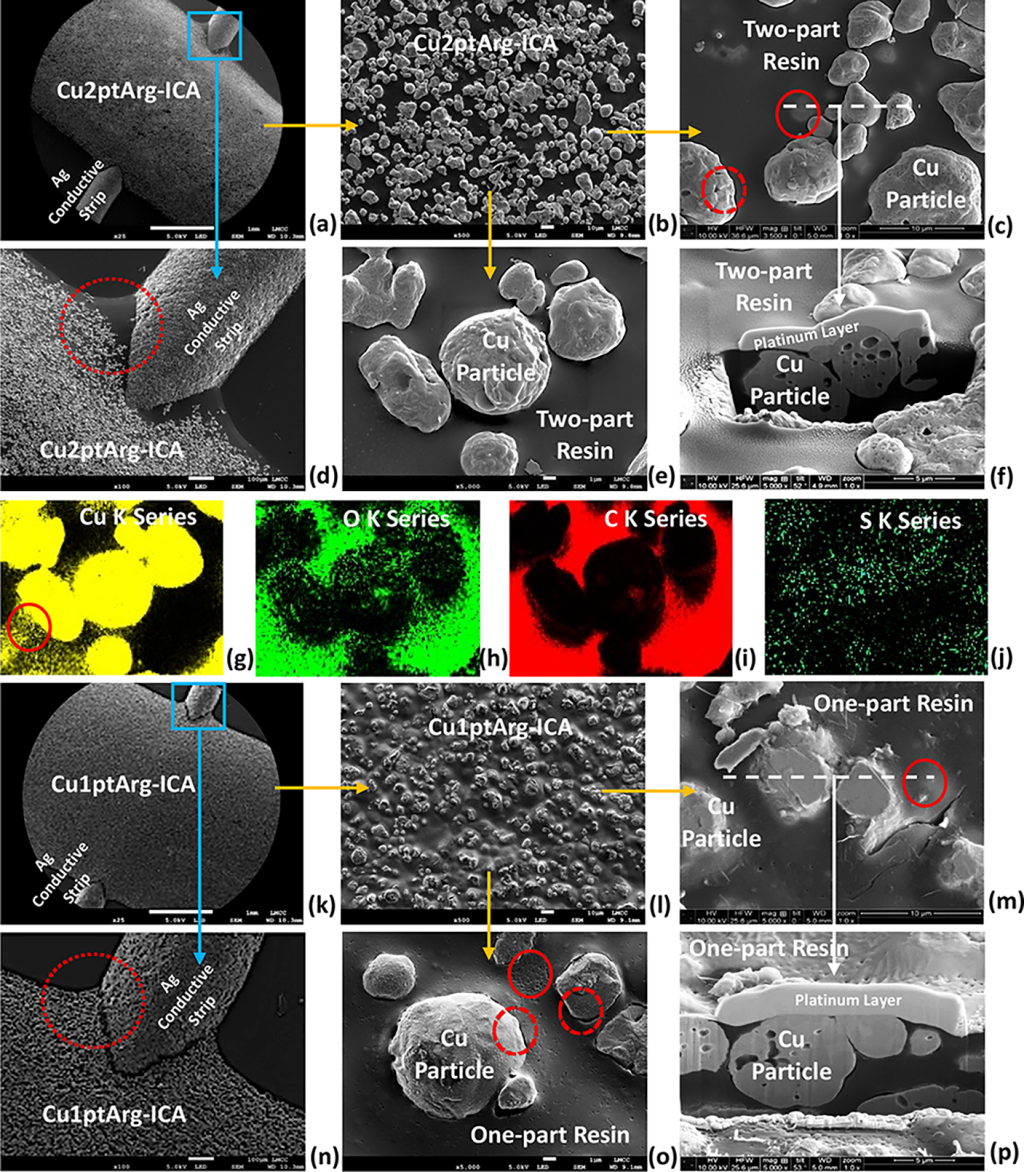
SEM analysis of (a–j) cured Cu2ptArg-ICA and (k–p) cured Cu1ptArg-ICA. (a–e, k–o) FEG-SEM images: (a, k) low and (d, n) higher magnification views of Cu-ICA tracks each crossing a Ag-ICA contact; (b, l) low and (c, e, m, o) high-magnification top-surface views of Cu-ICA tracks. (f, p) FIB-SEM images of Cu-ICA tracks after sectioning at the region indicated by the white dashed lines in panels c and m, respectively. (g–j) SEM-EDS-maps of the same region in top surface views of a Cu2ptArg-ICA track (panel e) (Figure 4a,b,f was adapted from [2018] IEEE Reprinted with permission from ref (36)).

Figure 4 shows FEG-SEM images of a range of cured ICA samples. Figure 4a,d,k,n shows one- and two-part Cu-ICA tracks crossing one of the preprinted Ag-ICA contacts. The electrical measurements showed that both types of Cu-ICAs made a good connection to the Ag-ICA contacts, and these images further demonstrate this. It can be observed that Cu particles were exposed at the surface of the cured ICA and appeared to be largely uniformly dispersed. However, around the joint between the Cu2ptArg-ICA track and Ag-ICA there is mostly resin visible at the surface (Figure 4d), seemingly due to separation of the two-part resin from the Cu particles during printing and curing. In comparison, the joint between the Cu1ptArg-ICA track and Ag-ICA had much less resin visible (Figure 4n), which is considered to result from the weight loss of the one-part resin during thermal curing and the resulting volume reduction.

Figure 4b,c,e,l,o shows higher magnification examples of the top surfaces of one- and two-part Cu-ICAs. It can be observed that the Cu particles appear relatively smooth, with only one or two pores in their surface, which is typical of the as-received condition of the powder. The two-part resin shows a smooth surface and fairly good contact with the Cu particles, and it is interesting to note that the resin has not covered them, but appears instead to have dewetted (Figure 4e), which is consistent with the XPS results (Figure 3h). The one-part resin shows a rougher surface and, in some areas, a small gap between the resin and Cu particles after curing (broken red circle in Figure 4o), due to shrinkage. However, the two-part resin, which is believed to have shrunk much less, also shows a small gap between the resin and particles (broken red circle in Figure 4c), indicating that the adhesion between the Cu and two resins was limited, most likely due to the presence of the low surface energy ODT-SAM. In contrast, for Cu-ICAs prepared from UT-Cu and AE-Cu, there was much less evidence of dewetting or exposure of the Cu particles at the surface of the ICA due to their higher surface energy (Supporting Information, S3).

Figure 4f,p shows FIB-SEM cross sections taken through Cu2ptArg-ICA and Cu1ptArg-ICA samples, respectively (indicated by the white dashed lines in Figure 4c,m). The solid red circles highlight buried Cu particles that are revealed by the FIB-SEM cross sections. In the cross sections, there appears to be direct surface contact between Cu particles in the cured resin, suggestive of the formation of a conductive network.

Figure 4g–j shows EDS-maps of the elemental distributions at the top surface of the Cu2ptArg-ICA sample shown in Figure 4e. Some Cu particles near the top surface of the ICA track are partly immersed in the resin, which can be seen in Figure 4e,g. Noticeably, the S signal (Figure 4j) is strongest where there is Cu, while O and C are mostly associated with the resin (Figure 4h,i).

### 3.4 TEM Investigation of Cured ICAs

The theoretical thickness of the ODT-SAM on Cu is ∼2.23 nm (not including the Cu–S bond),^35^ which is negligible when compared to the particle size. TEM characterization was therefore undertaken to investigate the particle-to-resin and particle-to-particle interfaces within the cured Cu-ICAs, using FIB to prepare 200 nm thick cross sections.

**Figure 5:**
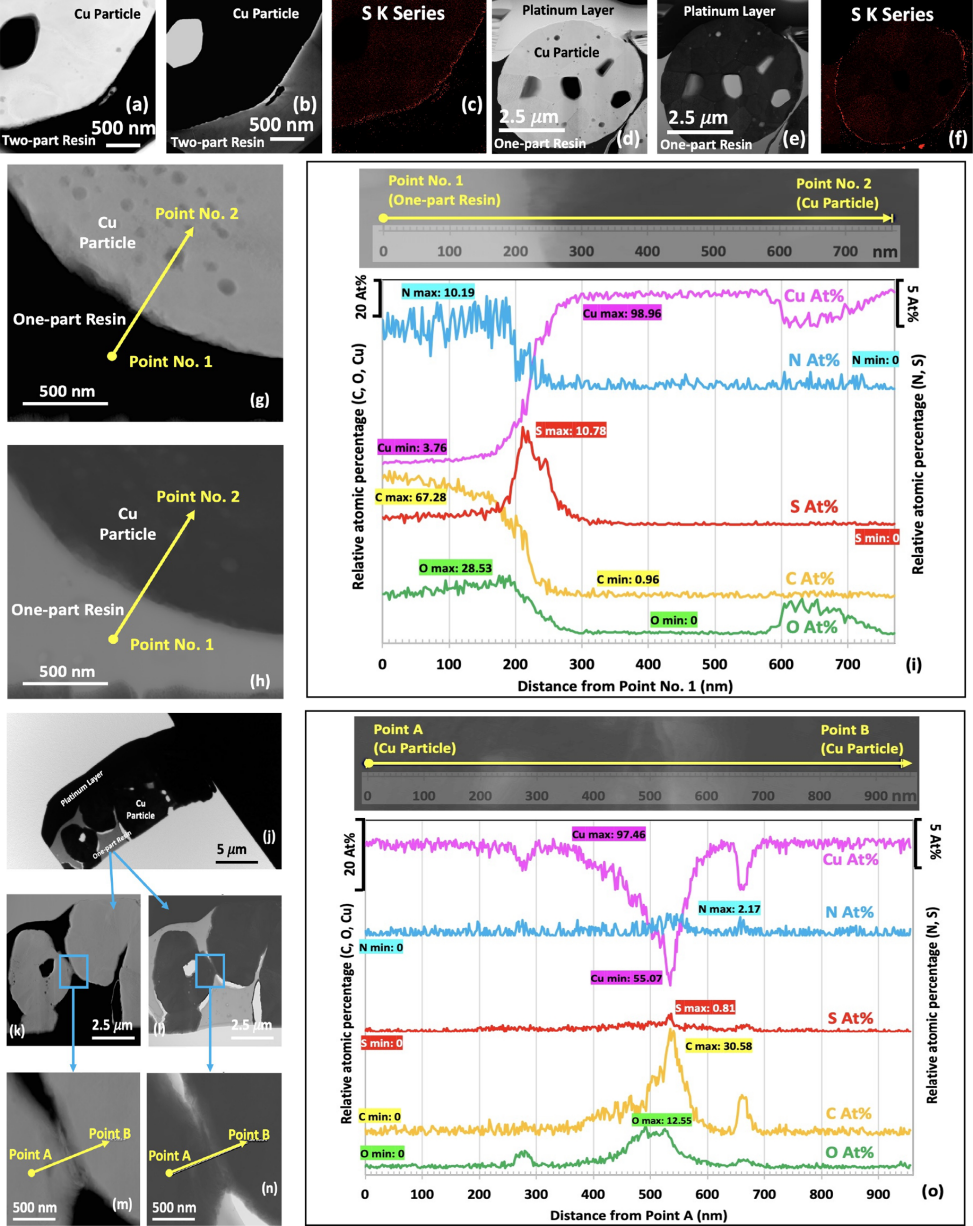
Scanning TEM dark-field and bright-field images and sulfur (S) EDS-maps of typical cross sections of freshly made Cu2ptArg-ICA (a–c) and Cu1ptArg-ICA (d–f) samples, respectively. Closer inspection of a cross section from a Cu1ptArg-ICA shows the interface between Cu and resin in dark-field (g) and bright-field (h) images. Panels j–n are two Cu particles in close contact viewed at increased magnifications, panels j, l, and n are STEM/bright-field images, and panels k and m are STEM/dark-field images. (i) Elemental distribution profiles across the particle-to-resin interface from Point No. 1 to Point No. 2 shown in panels g and h; and (o) elemental distribution profiles from particle-to-particle from Point A to Point B shown in panels m and n (Note: traces have been offset in the *y* direction for clarity, and different *y* axes have been used for the different elements).

Scanning TEM dark-field and bright-field images of typical cross sections of Cu2ptArg-ICA and Cu1ptArg-ICA are shown in Figure 5a,b;d,e, respectively. Crystal grains, grain boundaries within the Cu particles, and epoxy resins can be seen clearly by the diffraction contrast in bright-field images and Z-contrast in dark-field images. There is evidence of separation (bright contrast) in some places between the resin and Cu, and nanosize pores within the Cu particle can also be observed, in addition to the larger pores seen earlier by FIB-SEM (Figure 4f,p). The elemental distribution of the same regions was examined using STEM/EDS mapping, for which the results are shown in Figure 5c,f for Cu2ptArg-ICA and Cu1ptArg-ICA, respectively. It is notable that S intensity generally appears stronger around the outside of the Cu particle, which again indicates that some of the SAM remains on the particle surface after the thermal curing of ICAs, although Cu–S residue from breakdown of the SAM cannot be excluded.

TEM analyses of two regions from another cross section of a Cu1ptArg-ICA sample are shown in Figure 5g–o. In Figure 5i, the EDS line scan from Point No. 1 to Point No. 2 in Figure 5g,h shows the elemental distribution (At. %) across the particle/resin interface. The line scan spot size is ∼1 nm with a step interval of 2 nm. The interface between the Cu particle and one-part epoxy resin is located between 200 and 300 nm, where C and Cu show significant changes in intensity. At about 200–250 nm, an obvious S peak appears, further indicating the presence of SAM residues after thermal curing of the Cu-filled ICAs. The apparent width of the interface is ∼50 nm at full-width half-maximum of the peak, but this has been convoluted by the electron beam spot size and spread function of the beam through the thickness of the sample (∼200 nm), such that this is much wider than the theoretical thickness of an ODT-SAM.

The interface shown in Figure 5j–o indicates that a direct particle-to-particle contact could readily allow electron transfer. In particular, Figure 5m,n shows that the two Cu particles are connected to each other. In Figure 5o, the elemental intensity across the junction of the two Cu particles is investigated from Point A to Point B, as shown in Figure 5m,n. The position of the interface is at about 400–600 nm along the line scan. The Cu level decreases significantly, to about 50 At. % at around 535 nm, indicating that the two connected Cu particles were not initially joined together as a single larger particle and have moved into contact with each other during the mixing or curing steps. In the same region (400–600 nm), all of the other elements (notably S) show a peak value, indicating that, around the interface between the two Cu particles, there is a mixture of SAM residue and one-part epoxy resin.

## 4 Discussion

### 4.1 Contact Resistance and Surface Oxide Levels

**Figure 6:**
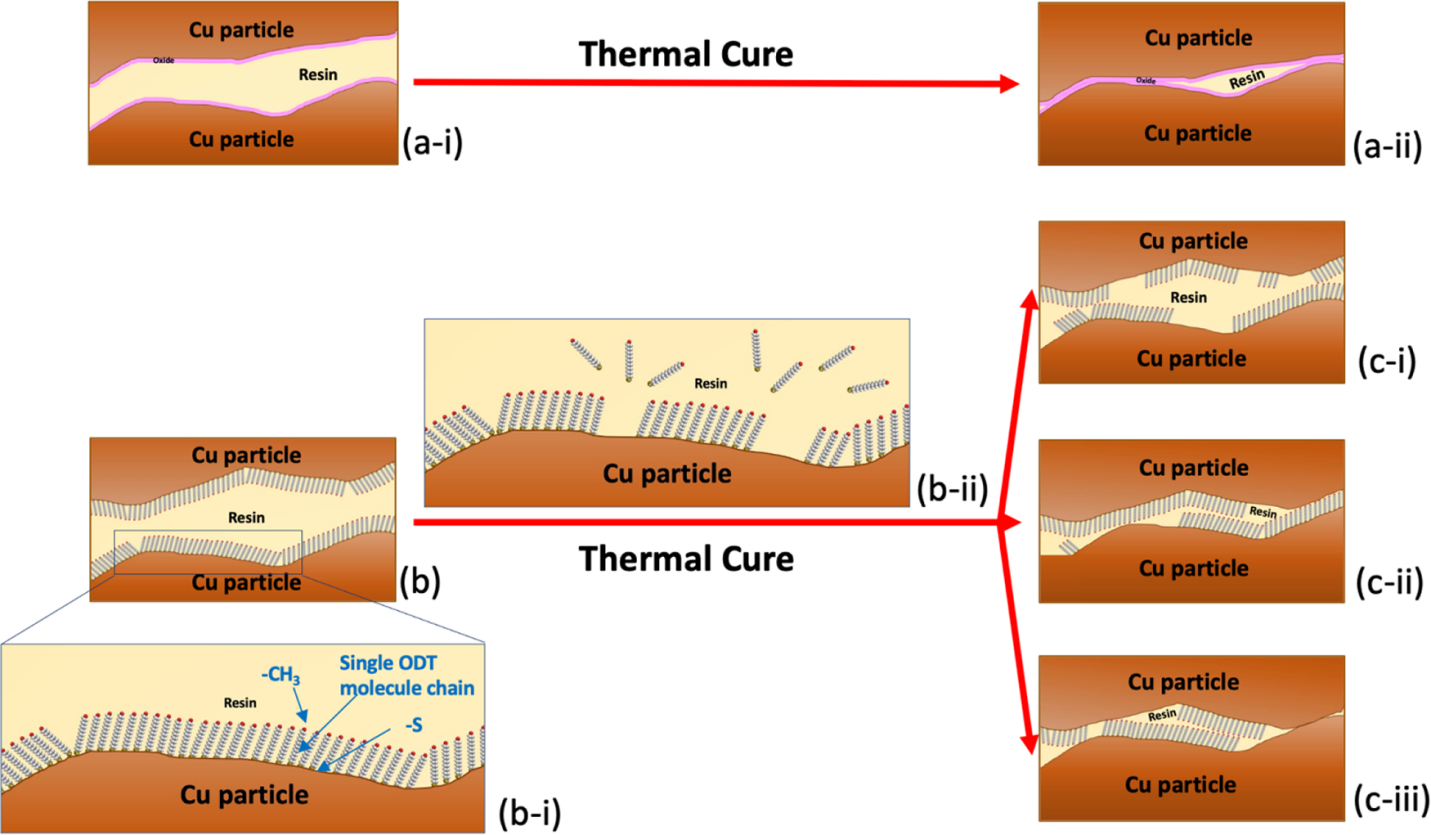
Schematic diagrams depicting the contact of two Cu particles in an ICA. (a) Without SAM before (a-i) and after (a-ii) thermal curing. (b) Approach of two ODT-SAM-Cu particles in an ICA before curing with enlargement of the SAM (b-i) and ODT desorption during thermal curing (b-ii). (c) Possible SAM involvement in the contact between two Cu particles in the cured ICA.

Electrical conduction in ICAs is achieved through the inclusion of a high volume fraction of conductive filler particles (Figure 2b), and the conduction mechanism can be explained by direct particle-to-particle contact or by electron tunneling through thin insulating layers, such as adhesive resin (<10 nm) to allow the transfer of electrons.^11,55−57^ Although high electric fields generated at short particle separations has also been proposed as a mechanism.^58^ Possible mechanisms for electrical conduction within the ODT-SAM-Cu-filled ICAs are proposed in Figure 6. The SEM (Figure 4) and TEM (Figure 5) investigations of the cured ICAs showed evidence of physical contact between the Cu particles. However, for high conductivity, these contacts must be of low resistance.^11^ The electrical path is affected by the thickness of any organic resin films between the particles^55−57,59,60^ and also the surface condition of the particles themselves, for example, the presence of high-resistivity oxides. In the ODT-SAM-Cu system presented here, the addition of an ODT-SAM onto the surface of the Cu particles also presents the possibility of additional interfacial layers that may affect conduction.^55−57,60^

From Figure 2, the high electrical conductivity of the cured Cu-ICAs made with ODT-SAM-Cu clearly demonstrates the effectiveness of the initial etching procedures and removal of the native oxides from the Cu. Short-term storage of the ODT-SAM-Cu in the freezer led to a small increase in surface oxide, but the Cu-ICAs made from these powders were still highly conductive. XPS (Figure 3) showed high levels of Cu(I) and Cu(II) oxides on the untreated Cu (UT-Cu), from which ICAs with poor conductivity were produced, while etched Cu (AE-Cu) with much lower oxide content could be used to make ICAs with low conductivity. In the latter case, even the presence of the relatively small amount of oxide that formed during short-term exposure of the AE-Cu to air was sufficient to limit the ICA conductivity significantly. Thus, the mechanism of two Cu surfaces without SAMs coming into contact within the ICA can be depicted, as shown in Figure 6a. The XPS data (Figure 3) indicate that the ODT-SAM deposition method followed here leads to a monolayer structure on the Cu particles, typical of other thiol on Cu, Au, and Ag systems^61^ (Figure 6b–i). A key aspect of the sample preparation used here is the initial ODT deposition (prerinse step) to make PR-Cu. This takes place during the final stages of ethanol rinsing as part of the filtration process after hydrochloric acid etching, and this process sequence avoids air exposure to the powder. In the results presented here, although very low levels of Cu(I) surface oxide form on the Cu below the SAM, they are expected to be electrically semiconductive,^62^ and therefore, with the presence of little or no Cu(II) surface oxide, a key requirement to enable the use of Cu particles in an ICA has been met, such that they are unlikely to present electrically resistive interfaces.

### 4.2 Comparison of One- and Two-Part Adhesives

Electrical conductivity of ICAs is often only achieved after curing of the adhesive matrix, which enables the fillers to form a high density of interconnections.^4,11^ Before curing, the two Cu-ICAs were not conductive, even though their weight contents were similar to those of the cured materials. Figure 6b shows a schematic view of the approach of two ODT-SAM-Cu particle surfaces before thermal curing with an interlayer of epoxy resin between them. The content of Cu_2_O in the freshly prepared samples was very low according to the XPS HR spectra (Figure 3), and it is not shown in the figure at this stage. During the curing process, the resin may be displaced by the approaching particles, so that they make contact, or may remain as a thin film between them.^2,11,55,57−59,63^

In this study, it was noticeable that ICAs made from the one-part resin showed higher electrical conductivity, for the same initial filler content, as compared with those made from the two-part resin. This was shown to be partly due to the difference in the Cu weight loading within the cured materials (Figure 2), but it did not completely explain the difference. Khairul Anuar et al.^46^ also saw a similar effect in Ag-filled one- and two-part ICAs and attributed this to greater shrinkage of the one-part system during cure. In the current study, the two-part resin showed very little or no weight loss and very little shrinkage upon curing, while the one-part resin showed significant weight loss and volume reduction. Higher shrinkage of the resin has also been shown in other studies to lead to increased conductivity of ICAs^3,29,30,33,57,60^ due to the reduction in thickness of interfacial layers between particles that reduces the tunneling resistance.^57,60,64^

### 4.3 Electrical Conduction and Interfacial Layers

The function of the SAM, and its fate during and after thermal curing, requires some discussion and several possible situations may occur at the particle interfaces, as suggested in Figure 6c-i–iii. In all cases, a residual layer of cured adhesive may remain in between the particles or it may be displaced by the approaching particles.^57,60,64^ One consequence of the low surface energy of the ODT-SAM may be that the resin can readily flow out of the area between the particles as they approach and as the adhesive around the particles shrinks during cure. The ability of the resin to flow off the Cu particles is demonstrated by their exposure at the surface of the cured tracks by SEM (Figure 4) and XPS (Figure 3). Furthermore, the small gaps sometimes observed between the Cu particles and resin (Figures 4 and 5) suggest weak adhesion between them, which is also likely to be caused by the low surface energy of the ODT-SAM. SAMs with, for example, amine terminal groups have been used to enhance adhesion between epoxy and Cu and could therefore be investigated in the future with regard to their oxidation preservation characteristics and influence on ICA reliability.^65^ Furthermore, the surface energy of the terminal group is likely to influence the interaction of the filler with the resin, affecting the rheology of the resulting paste similar to that of lubricant films on Ag flake fillers.^56,59^

The S present in the original ODT-SAM gave low intensity XPS peaks, but its continued presence in the XPS spectra from the cured ICAs further supports the model that the adhesive flowed off the particle surfaces, enabling the weak S signal to be detected. It also supports the theory that part of the SAM remains on the Cu surface. The TEM observations (Figure 5) also showed evidence of S on the surfaces of the particles within the cured ICAs. The loss of some SAM from the Cu surface during short-term heating to 150 °C is suggested by the XPS data of the heat-treated powder, which included evidence of increased uptake of O on exposure to air during transfer to the instrument. The thermal stability of SAMs on Cu has been reported in the literature. In the most recent investigation, Ito et al.^52^ found that octanethiol on polycrystalline Cu showed low levels of desorption as disulfides at around 390 K followed by greater desorption of thiols and thiolates from 430 to 450 K and proposed a mechanism of complete molecule desorption from the surface. In contrast, Carbonell et al.^54^ proposed that oxidized sulfur from decanethiol desorbed from metallic Cu surfaces by C–S bond scission reaching a maximum around 95 °C with the remaining alkyl chains desorbing above this temperature toward a maximum around 150 °C. Interestingly, the peak desorption temperatures identified in the works of Ito and Carbonell are similar but differ in their interpretation of the desorbing species. Sung et al.^53^ found that octanethiol SAMs on Cu(111) were stable to around 200 °C but then started to degrade by C–S bond scission during thermal desorption in ultrahigh vacuum to leave behind Cu_2_S. Sung and Kim^66^ also found that hexadecanethiol SAMs on Cu were stable to around 140 °C and completely desorbed around 180 °C. The desorption process appears to depend sensitively to the temperature, level of surface oxide present, chain length, and desorption environment. The longer chain ODT molecules used in this study are likely to have more thermal stability compared to those mentioned in the above studies but are expected to behave in a similar fashion. Remaining ODT molecules on the Cu surface may be randomly dispersed, or remain as islands, although lying down structures have also been observed on Au surfaces.^52^

Based on the previous discussions, a number of possible models for the role of the SAM during particle-to-particle contact and electrical conductivity within the ICA are proposed, as shown in Figure 6c-i–iii. As mentioned earlier, these schematic diagrams assume the displacement of adhesive from between the particles, facilitated by any remaining low surface energy SAM and resin shrinkage (however, its presence cannot be ruled out). The underlying Cu is also assumed to remain largely oxide free, due to the Ar curing atmosphere and barrier to oxygen provided by the encapsulating resin. In Figure 6c-i–iii, the SAM is assumed to remain as islands on the Cu surface, following desorption (Figure 6b-ii) of some ODT molecules or break down of chains into the surrounding resin during the thermal curing step. Where the particles contact, the SAM may be on both or just one surface (Figure 6c-i,ii). These islands may still prevent direct metal–metal contact. In this case, electrical conduction will take place via tunneling through the SAM, or its electrical breakdown.^11,55,57,58,60,67−69^ The role of the SAM in this case is to maintain the largely oxide free condition of the underlying Cu during mixing and processing, and its absence may limit the longer term reliability of the cured material. Alternatively, there may be sufficient removal or thinning of the SAM that the metal surfaces move into direct contact, assisted by the resin shrinkage (Figure 6c-iii). In this case, the contact resistance is dependent on the area of contact and on the resistance of any residual oxide layers. It is also possible that the extended curing time of the one-part resin compared to the two-part resin could lead to greater thinning of the SAM layer for the one-part ICAs, leaving less material between the particles and increasing the conductivity.^55−57,59^

## 5 Conclusions

The aim of this work was to examine the function of the ODT-SAM in the protection of micrometer-scale Cu powder and its role in the electrical conduction of thermally cured Cu-ICAs. The effect of the SAM deposition process steps in the etching of pre-existing surface oxides from Cu powder and the subsequent deposition of SAMs of ODT was shown to lead to low levels of surface oxidation. One- and two-part epoxy ICAs were prepared with these ODT-SAM-Cu powders as fillers and, after thermal curing in an Ar atmosphere, produced tracks with electrical conductivity matching that of commercial Ag-filled adhesive. ICAs prepared from one-part epoxy gave higher conductivity, which could only be partially attributed to epoxy weight loss, and the shrinkage of the material is thought to play an important role in improving the conductivity. Assessment of ICAs made from Cu taken at different stages of the etching and SAM deposition process demonstrated that the surface oxide level of the Cu powder had a significant effect on the electrical conductivity of the cured ICAs, with untreated or unprotected Cu powders resulting in very poor conductivity. Thermally cured Cu-ICAs still showed the presence of S at the surface of the Cu particles in XPS analysis, with only low levels of Cu oxide noted. In addition, STEM analysis of the interfaces of particles within the cured ICA revealed the presence of S. The density or coverage of the ODT-SAM is thought to be reduced by the thermal curing process, but further investigation is required to determine the details of SAM degradation. From the observations presented, the mechanism of conduction between particles can be generally explained by either direct Cu-to-Cu contact to allow the transfer of electrons and/or through electron transport by tunneling through any remaining ODT-SAM, or a very thin layer of adhesive resin. This research highlights the role of ODT-SAMs in enabling the use of Cu in electrically conductive adhesives and demonstrates their potential as lower cost and environmental impact alternatives to existing interconnect materials.

## Supporting Information Available

Copper particle size distribution and SEM images, influence of the storage time on the SAM-coated Cu powder surface composition and resulting ICA conductivity, and structure of ICAs made from non-SAM-coated Cu powder (PDF)
