# Supplementary material for: The Role of Self-Assembled Monolayers in the Surface Modification and Interfacial Contact of Copper Fillers in Electrically Conductive Adhesives
Source: ACS Appl Mater Interfaces. 2023 Dec 19;16(1):1846–60. doi: 10.1021/acsami.3c14900 (PMC10788863; doi:10.1021/acsami.3c14900)
Supplement: Supplementary file 1 — am3c14900_si_001.pdf [file am3c14900_si_001.pdf]

Supporting Information

The Role of Self-Assembled Monolayers in the Surface Modification and Interfacial Contact of Copper Fillers in Electrically Conductive Adhesives

Shanda Wang[1,3*], Zhaoxia Zhou[2], Athanasios Goulas[2], Gary W. Critchlow[2], David C. Whalley[1] and David A. Hutt[1*]

[1] Wolfson School of Mechanical, Electrical and Manufacturing Engineering, Loughborough University, Loughborough, Leicestershire, LE11 3TU, UK

[2] Department of Materials, Loughborough University, Loughborough, Leicestershire, LE11 3TU, UK

[3] Present address – State Key Laboratory of Chemical Safety, SINOPEC Research Institute of Safety Engineering Co., Ltd., Qingdao, Shandong, 266101, China

[*] Corresponding authors email: D.A.Hutt@lboro.ac.uk; S.Wang5@lboro.ac.uk; wangsd.qday@sinopec.com

S1 Copper Particle Size Distribution

The electrically conductive filler particle shape and size distribution can play an important part in the conductivity of isotropic conductive adhesives (ICAs)[1,2]. The average particle size by volume of the powder used in the present study was specified by the supplier to lie between 14-25 µm. **Figure S1(a) & (b)** show scanning electron microscopy (SEM) images of the copper (Cu) powder used in this study, before (untreated, **Figure S1(a)**) and after (**Figure S1(b)**) the deposition of an octadecanethiol (ODT) self-assembled monolayer (SAM) using the process described in the main paper. The particles were generally spheroidal in nature and included some pores in the surface. Overall, the particles were well distributed and separated from each other. There was no apparent difference between the particles before and after SAM deposition.

The particle size distribution of the powder was determined from the SEM images using ImageJ software[3] to measure manually the diameter of around 460 particles. **Figure S1(c)** shows the distribution of the particle sizes by number percent, i.e., the percentage of the total number of particles that were of a particular diameter ±0.5 µm. From the measured distribution, the average particle size, based on the number percent of particles, was found to be 9.2 µm. This number is

similar to that obtained using laser diffraction from a different batch of powder with the same product code[4]. The difference in the average particle size determined from the number percent distribution compared to volume percent distribution is due to the interpretation methods of the data that are influenced by the higher volume occupied by large particles compared to small particles[5].

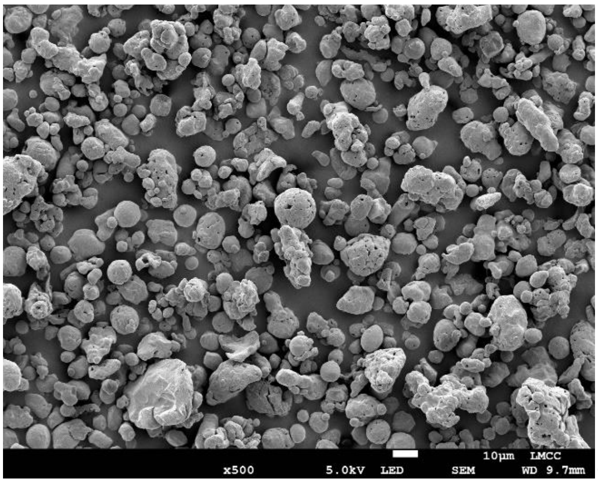

(a)

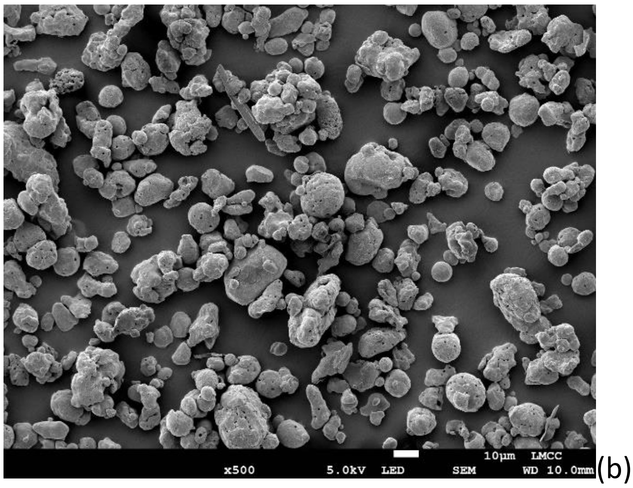

(b)

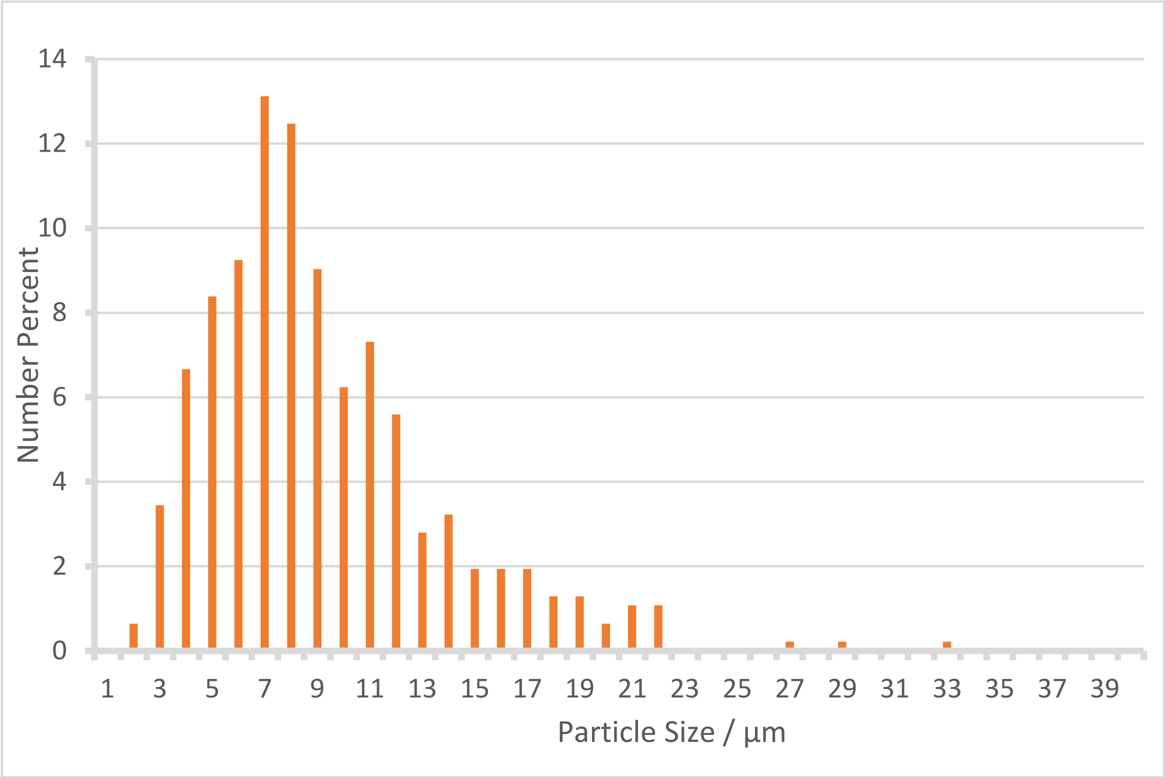

(c)

Figure S1. Example SEM images of (a) untreated (UT-Cu) and (b) SAM coated (ODT-SAM-Cu) powder samples. (c) Histogram distribution of particle size by number percent obtained using ImageJ analysis.

S2 Effect of storage time in air in the freezer on ODT-SAM-Cu

After etching and SAM deposition onto the Cu powder surface, following the methods described in the main paper, the ODT-SAM-Cu powders were contained in glass jars with the ambient atmosphere sealed inside and then stored in a freezer until required for use. On removal from the freezer, they were allowed to reach room temperature before the lid was removed and the powder weighed and mixed with one- or two-part epoxy resin to form a Cu-ICA.

The effect of the storage time in the freezer before use of the powders was investigated. **Table S1** shows the XPS surface composition analysis of powders stored for 0 and 30 day(s) in the freezer. These were from the same batch prepared on the same occasion. It can be seen that very little changed in the composition during this storage time at low temperature, consistent with other work on the use of SAMs for oxidation protection in the literature[4,6–8].

In addition to the XPS results, **Table S1** also shows the electrical conductivity of one-part Cu-ICAs prepared from the same powder stored for 0, 15 and 30 days. The data for 15 days storage is the same as that presented as Example 2 in Figure 2(a) of the main paper. There was very little change in the conductivity over this storage period, consistent with the XPS analysis.

As an example of the long-term stability of the ODT-SAM-Cu powders when stored in a freezer, **Table S1** also shows electrical conductivity data for ICAs made from a different batch of powder stored for over 1000 days. Both the 1pt and 2pt ICAs prepared from these materials showed good conductivity despite this long-term exposure to air.

Table S1. XPS composition analysis and resulting Cu-ICA electrical conductivity for ODT-SAM-Cu with different freezer storage times. (TSM and LSM refer to the Top Surface and Lower Surface Conductivity Measurements from the printed tracks respectively, as described in the main paper).

Freezer Storage Time (Days)	XPS Composition Analysis (At% of elements)					ICA Preparation	ICA Electrical Conductivity / 10^5 Sm^{-1}
	Cu	O	C	S	Cl		
0	16.08	1.98	76.41	4.06	1.47	Cu1ptArg-ICA	18.91 $\pm$ 0.93 (TSM)<br>18.97 $\pm$ 1.18 (LSM)
15	N/A	N/A	N/A	N/A	N/A	Cu1ptArg-ICA	18.80 $\pm$ 1.96 (TSM)<br>18.89 $\pm$ 1.86 (LSM)
30	16.44	2.10	75.83	3.92	1.71	Cu1ptArg-ICA	18.68 $\pm$ 1.91 (TSM)<br>18.75 $\pm$ 2.08 (LSM)
>1000	N/A	N/A	N/A	N/A	N/A	Cu1ptArg-ICA	14.90 $\pm$ 3.22 (TSM)<br>14.98 $\pm$ 2.96 (LSM)
>1000	N/A	N/A	N/A	N/A	N/A	Cu2ptArg-ICA	5.55 $\pm$ 0.81 (TSM)<br>5.65 $\pm$ 1.15 (LSM)

S3 Structure of Cu-ICAs made from UT-Cu and AE-Cu

Cu particles that did not receive an ODT SAM coating, i.e., untreated Cu (UT-Cu) and Cu that had only been etched with HCl and rinsed with ethanol (AE-Cu) were also used to prepare 1pt and 2pt Cu-ICAs using the same formulations as the SAM coated materials. **Figure S2** shows SEM images of the top surfaces of thermally cured one-part Cu-ICAs made of UT- and AE-Cu. In contrast to the ODT-SAM-Cu ICAs where de-wetting of the particles was evident (Figures 4(b,c,e,l,m,o) in the main paper), for the UT-Cu ICA (**Figure S2(a,b)**) there is evidence of the resin still largely surrounding the Cu particles that have aggregated into clumps. For the AE-Cu-ICA (**Figure S2(c,d)**), the particles are immersed in the surrounding resin with very little of the Cu exposed. This implies that the particles still maintain a layer of resin between them that is likely to affect the resistance of the contacts in addition to any oxidation of the Cu.

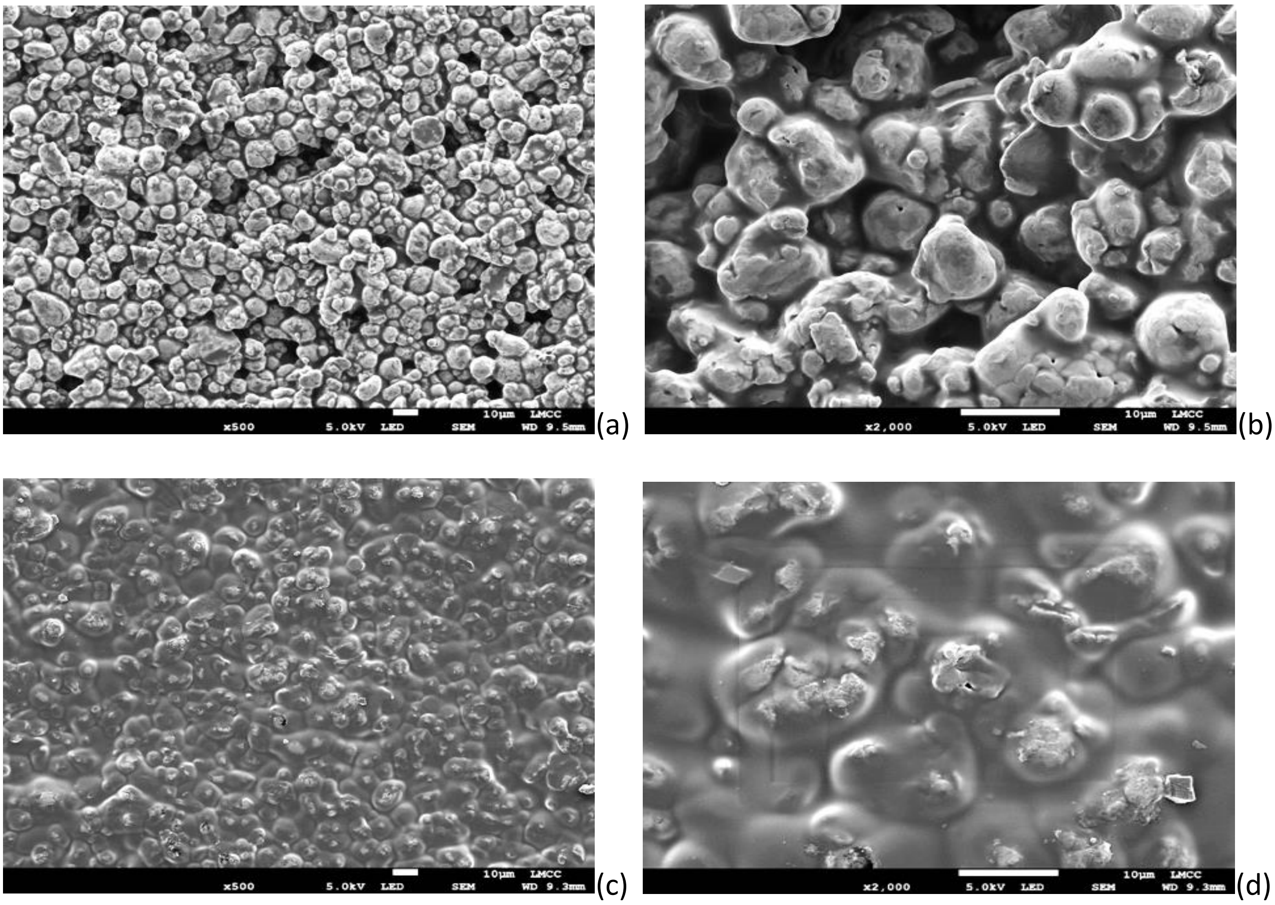

Figure S2 SEM images of the surfaces of thermally cured Cu 1pt ICAs prepared with (a,b) UT-Cu and (c,d) AE-Cu.

References

(1) Yim, M. J.; Li, Y.; Moon, K. S.; Paik, K. W.; Wong, C. P. Review of Recent Advances in Electrically Conductive Adhesive Materials and Technologies in Electronic Packaging. *Journal of Adhesion Science and Technology*. September 1, 2008, pp 1593–1630. https://doi.org/10.1163/156856108X320519.

(2) Khairul Anuar, S.; Mariatti, M.; Azizan, A.; Chee Mang, N.; Tham, W. T. Effect of Different Types of Silver and Epoxy Systems on the Properties of Silver/Epoxy Conductive Adhesives. *Journal of Materials Science: Materials in Electronics* **2011**, *22* (7), 757–764. https://doi.org/10.1007/s10854-010-0207-7.

(3) *ImageJ Software*. https://imagej.net/ij/index.html (accessed 2023-09-13).

(4) Qi, S. Microwave Assisted Processing of Metal Loaded Inks and Pastes for Electronic Interconnect Applications. Doctoral Thesis, Loughborough University, 2014. https://hdl.handle.net/2134/16118 (accessed 2023-09-12).

(5) Shimadzu. *Volume Standard and Number Standard in Particle Size Distribution*. https://www.shimadzu.com/an/service-support/technical-support/analysis-basics/lesson17.html (accessed 2023-10-04).

(6) Laibinis, P. E.; Whitesides, G. M. Self-Assembled Monolayers of n-Alkanethiolates on Copper Are Barrier Films That Protect the Metal against Oxidation by Air. *J Am Chem Soc* **1992**, *114* (23), 9022–9028. https://doi.org/10.1021/ja00049a038.

(7) Hutt, D. A.; Liu, C. Oxidation Protection of Copper Surfaces Using Self-Assembled Monolayers of Octadecanethiol. *Appl Surf Sci* **2005**, *252* (2), 400–411. https://doi.org/https://doi.org/10.1016/j.apsusc.2005.01.019.

(8) Qi, S.; Vaidhyanathan, B.; Hutt, D. Conventional and Microwave-Assisted Processing of Cu-Loaded ICAs for Electronic Interconnect Applications. *J Mater Sci* **2013**, *48* (20), 7204–7214. https://doi.org/10.1007/s10853-013-7537-9.
